# Association of thyroid dysfunction and COVID-19: A systematic review and meta-analysis

**DOI:** 10.3389/fendo.2022.947594

**Published:** 2022-10-28

**Authors:** Mohammad Darvishi, Mohammad Reza Nazer, Hamze Shahali, Majid Nouri

**Affiliations:** ^1^ Infectious Diseases and Tropical Medicine Research Center (IDTMRC), Department of Aerospace and Subaquatic Medicine, AJA University of Medical Sciences, Tehran, Iran; ^2^ Depertment of Infectious Diseases, Isfahan University of Medical Sciences, Isfahan, Iran; ^3^ Aerospace and Sub-Aquatic Medical Faculty, Aja University of Medical Sciences, Tehran, Iran; ^4^ Infectious Diseases and Tropical Medicine Research Center (IDTMRC), AJA University of Medical Sciences, Tehran, Iran

**Keywords:** COVID-19, thyroid, TSH, T3, T4, severity

## Abstract

This systematic review and meta-analysis was conducted to evaluate the effect of COVID-19 on thyroid function and the role of thyroid hormones alterations in predicting the severity of COVID-19. Online databases, including Scopus, Medline/PubMed, EMBASE, Google Scholar, and Cochrane were searched up to August 2, 2022. After screening titles, abstracts, and full manuscripts, respectively, 30 reports were enrolled. The risk of bias (ROB) was evaluated using the QUADAS-2 tool. In addition, odds ratio (OR) and hazard ratio (HR) analysis for assessing the OR of abnormal thyroid function tests (TFT) in predicting the COVID-19 severity and poor outcomes. Among 30 enrolled studies, ROB of the current study is estimated low to moderate. The average number of patients in each study was 325 (range: 40-3,703), with an overall mean age of 57.6, and the female proportion of 40.4%. Overall, the pooled analysis showed that the prevalence of thyroid dysfunction among 9,707 COVID-19 cases was 15%. Among mild to moderate COVID-19 patients, 6.2% had abnormal TFT, and among patients who experienced severe to critical COVID-19, 20.8% had abnormal TFT. The pooled OR for abnormal TFT and the severity of COVID-19 obtained from 3,865 COVID-19 patients was 3.77 (2.03, 6.99). The pooled HR of TSH level of COVID-19 mortality was 1.57 (0.91, 2.72). Our results demonstrate a high prevalence of thyroid dysfunction in COVID-19, and that among patients severe cases had a 3.77-fold higher risk of abnormal TFT compared to mild to moderate COVID-19. Further studies are required to evaluate the longer-term prognostic role of thyroid dysfunction in severe COVID-19, and investigate potential therapeutic strategies.

## Introduction

Coronavirus Disease 2019 (COVID-19) is a new millennium pandemic with unprecedented public health challenges (1). The causative agent is a novel enveloped β-coronavirus called severe acute respiratory syndrome coronavirus 2 (SARS-CoV-2) (2). Since it was first detected in Wuhan, COVID-19 has spread rapidly, and outbreaks are increasing exponentially. As of December 28th, 2020, the number of patients afflicted with SARS-Cov-2 has surpassed 80M cases worldwide, and more than 1.7M people have now died of COVID-19. SARS-Cov-2 has a phylogenetic resemblance to SARS-CoV-1 and is related to SARS-CoV-1. SARS-CoV-2 infects human tissues entering cells through the angiotensin-converting enzyme 2 (ACE2) receptor (3).

COVID-19 can range from asymptomatic manifestations to severe and even fatal respiratory disorders (4). In high-risk patients (i.e., elderly or patients with a history of cardiovascular diseases, chronic hypertension, and diabetes), SARS-CoV-2 infection can induce both system and pulmonary inflammation (5). The most frequent serious complications of COVID-19 are acute respiratory distress syndrome (ARDS), respiratory failure, sepsis, acute cardiac injury, and heart failure (5).

The international scientific community has now reacted massively to the pandemic conquest of COVID-19, and a substantial number of studies have been carried out on different aspects of the disease, including prevention, diagnosis, and treatment. These vast investigations on COVID-19 has led to a growing body of evidence regarding a remarkable association between the prevalence of thyroid disorders in patients with COVID-19 (6).

A complex association has been documented between hormones and immunomodulatory signaling molecules in thyroid and viral infections (7). Viruses and their related inflammatory-immune responses are particularly noteworthy since they were noted to affect thyroid function permanently in some cases (8). Although several studies have evaluated the thyroid gland function in COVID-19, no comprehensive meta-analysis was conducted on this crucial topic so far. Thus, herein, we aimed to systematically evaluate the association between COVID-19 and thyroid function and the potential of thyroid hormones in predicting the severity of COVID-19 and present a meta-analysis of the current related data.

## Methods

### Searching strategy

Electronic databases, including Scopus, Medline/PubMed, EMBASE, Google Scholar, and Cochrane database were screened until August 2, 2022. We conducted a comprehensive search of PubMed and MEDLINE articles using the combination of the search terms “thyroid” and “coronavirus” (or “SARS-CoV-2” or “COVID-19”). An English language limitation was added. This study was carried out based on Desired reporting products for the Systemic and Meta-Analysis Review (PRISMA) (9).

### Study selection

Two blind reviewers conducted the title-abstract screening of all selected research separately. Full-text assessments were conducted afterward. Duplicate and unrelated reports were excluded at the title-abstract screening level before reviewing the full manuscripts.

### Inclusion criteria

All studies evaluating or mentioning the number of patients with abnormal thyroid function in COVID-19 cases were enrolled.

### Exclusion criteria

Molecular reports, laboratory observations in percentages, case studies, and statements were excluded.

### Data extraction

Two reviewers extracted the data independently, taking into account main attributes, including author, year of publication, method of analysis, sample size, lab observations, co-morbidities, and final clinical results.

### The assessment of methodological quality and risk of bias

The quality appraisal checklist and the critical appraisal methodological index for non-randomized studies were used for bias risk assessment. The revised “Quality Assessment of Diagnostic Accuracy Studies” (QUADAS) tool was used for the quality assessment of the included studies. This choice was based on former studies that have confirmed and suggested its utility for quality assessment of diagnostic studies in all domains of patient selection, risk of bias, reference standard, and flow and timing. Therefore, we have proceeded with this tool based on former validations (10). Moreover, the funnel plot and Egger’s regression test were used to assess publication bias (11).

### Statistical analysis

Cochran, Chi-Square, and I2 were used to determine the heterogeneity of tests. When I^2^ was more than 50%, a random-effects model was preferred to a fixed-effects model [15]. Also, we used the prevalence formula as below to evaluate the prevalence of abnormal thyroid test function (TFT) in COVID-19 cases:


$$\text{Prevalence=}\frac{Number\;of\;COVID-19\;cases\;with\;abnormal\;TFT}{Total\;number\;of\;COVID-19\;patients}$$

In addition, we used odds ratio (OR) and hazard ratio (HR) analysis to evaluate the association of abnormal TFT on the prediction of COVID-19 severity. A P-value less than 0.05 was considered statistically meaningful (2-sided). All data have been analyzed with R (version 4.0.2; R Foundation for Statistical Computing) and R studio (Version 1.3.1073).

## Results

### Study selection

**Figure 1:**
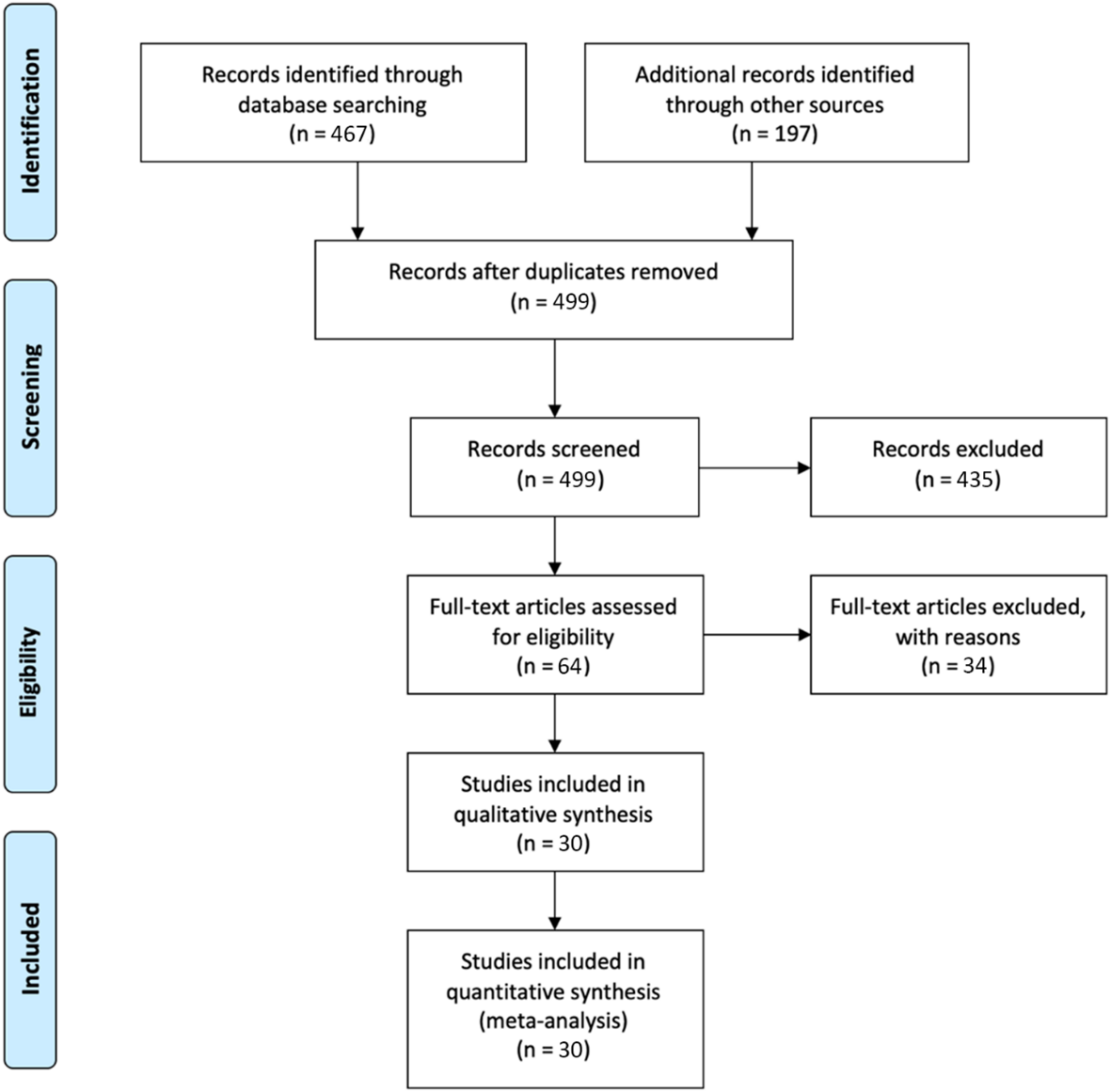
PRISMA flow diagram of study selection.

After removing duplicates, the initial literature search yeilded a total of 499 published records from PubMed, EMBASE, Google Scholar, Web of Science, and Scopus. The titles and abstracts were screened, and 435 records were excluded. Then we checked and reviewed the full texts of 64 remaining articles for evaluating eligibility based on the PRISMA algorithm (**Figure 1**). Eventually, 30 studies were deemed eligible for final inclusion. **Figure 1** illustrates the selection process of enrolled studies in a flow diagram.

Most of these publications included a series of expert opinions and suggestions on new methods for treating thyroid disorders in the face of the possibility of transmission of COVID-19 and the potential of health care spikes (5, 12–25). However, all 30 articles looked at thyroid activity or identified thyroid disorders in COVID-19 patients (5, 12–40). Twenty-three studies studied the TSH level and thyroid gland hormones, including fT3 and fT4, based on COVID-19 severity (5, 14–16, 19, 20, 23–26, 28, 31, 33, 35–38, 40); and nineteen studies evaluated the exact levels of TSH and thyroid gland hormones (12, 13, 17, 18, 20, 26–35, 37–40). Finally, four studies estimated the hazard ratio (HR) in COVID-19 survivors and non-survivors based on thyroid function (22–24, 40).

### Study characteristics

The main characteristics of the 30 included studies are summarized in **Table 1**. Twelve studies originated in China (12, 13, 15, 19, 20, 22, 24, 32, 38–40), four from Italy (21, 27, 30, 36), two from Hong Kong (17, 35), two from Spain (22, 28), and one each from Brazil, France, Greece, India, Kuwait, Saudi Arabia, South Korea, Turkey, United Kingdom, and the United States (14, 25, 26, 29, 31, 33, 34, 37, 41, 42). All included studies stated that RT-PCR confirmed the diagnosis of COVID-19 through typical COVID-19 symptoms except one study; thus, all studies enrolled symptomatic patients, and only one enrolled asymptomatic COVID-19 patients (43). Data were recorded retrospectively in all studies except five that stated prospective data collection (28, 33–36). All patients were enrolled consecutively. The average number of patients in each study was 325 (range: 40-3,703), with an overall mean age of 57.6, and the female proportion of 40.4% (**Table 1**).

**Table 1:** The main characteristics of 30 studies from different countries with RT-PCR confirmed the diagnosis of COVID-19.

Author	Design	Patients enrollment	Country	Final total No.	Male/Female	Mean age total	Mean age total SD	Patients COVID-19 diagnose	Index	Main concept	Abnormal Thyroid
Lui et al., 2020 (20)	Retrospective	Consecutive	Hong Kong	191	99/92	53.5	17.2	Symptomatic	RT-PCR	Evaluated thyroid function in COVID-19 patients with mild to moderate severity.	25
Chen, T et al., 2019 (5)	Retrospective	Consecutive	China	274	171/103	62	28.1	Symptomatic	RT-PCR	Clinical characteristics of severely or critically ill COVID-19 patients	49
Chen, M et al., 2020 (15)	Retrospective	Consecutive	China	50	30/20	48.4	13.7	Symptomatic	RT-PCR	Evaluated thyroid function in COVID-19 patients	32
Gao et al., 2020 (16)	Retrospective	Consecutive	China	100	52/48	62.8	13.6	Symptomatic	RT-PCR	Evaluated thyroid function in COVID-19 patients with mild to moderate severity.	15
Georges et al., 2020 (17)	Retrospective	Consecutive	France	433	259/174	63.9	17.1	Symptomatic	RT-PCR	Clinical characteristics of severely or critically ill COVID-19 patients	43
van Gerwen et al., 2020 (23)	Retrospective	Consecutive	United States	3703	2049/1654	58.1	18.2	Symptomatic	RT-PCR	Clinical characteristics of COVID-19 in patients with thyroid dysfunction	251
Cao et al., 2020 (14)	Retrospective	Consecutive	China	198	101/97	50.1	16.3	Symptomatic	RT-PCR	Clinical characteristics of severely or critically ill COVID-19 patients	6
Shabrawishi et al., 2020 (21)	Retrospective	Consecutive	Saudi Arabia	150	90/60	46.1	15.1	Symptomatic	RT-PCR	Clinical characteristics of severely or critically ill COVID-19 patients	9
Sisó-Almirall et al., 2020 (22)	Retrospective	Consecutive	Spain	322	161/161	56.7	17.8	Symptomatic	RT-PCR	Clinical characteristics of severely or critically ill COVID-19 patients	14
Wang et al., 2020 (24)	Retrospective	Consecutive	China	55	22/33	49	29.7	Asymptomatic	RT-PCR	Clinical characteristics of severely or critically ill COVID-19 patients	1
Yan et al., 2020 (25)	Retrospective	Consecutive	China	168	81/87	51	16.3	Symptomatic	RT-PCR	Clinical characteristics of severely or critically ill COVID-19 patients	2
Zhang et al., 2020 (12)	Retrospective	Consecutive	China	140	71/69	57	21	Symptomatic	RT-PCR	Clinical characteristics of severely or critically ill COVID-19 patients	5
Almazeedi et al., 2020 (13)	Retrospective	Consecutive	Kuwait	1096	888/208	41	16	Symptomatic	RT-PCR	Clinical characteristics of severely or critically ill COVID-19 patients	25
Lania et al., 2020 (18)	Retrospective	Consecutive	Italy	287	193/94	66	31	Symptomatic	RT-PCR	Clinical characteristics of COVID-19 in patients with thyroid dysfunction	73
Li et al., 2020 (19)	Retrospective	Consecutive	China	40	N/R	43.8	12.8	Symptomatic	RT-PCR	Clinical characteristics of severely or critically ill COVID-19 patients	N/R
Ahn et al., 2021 (26)	Retrospective	Consecutive	South Korea	119	62/57	64.3	16.8	Symptomatic	RT-PCR	Evaluated thyroid function in COVID-19 patients	43
Baldelli et al., 2021 (27)	Retrospective	Consecutive	Italy	66	34/32	60.8	17	Symptomatic	RT-PCR	Clinical characteristics of severely or critically ill COVID-19 patients	28
Ballesteros Vizoso et al., 2021 (28)	Prospective	Consecutive	Spain	78	55/23	59	12	Symptomatic	RT-PCR	Clinical characteristics of severely or critically ill COVID-19 patients	42
Beltrão et al., 2021 (29)	Retrospective	Consecutive	Brazil	245	100/145	62	NA	Symptomatic	RT-PCR	Evaluated thyroid function in COVID-19 patients	16
Campi et al., 2021 (30)	Retrospective	Consecutive	Italy	144	97/47	68.1	14.67	Symptomatic	RT-PCR	Clinical characteristics of severely or critically ill COVID-19 patients	56
Dutta et al., 2021 (31)	Retrospective	Consecutive	India	236	159/77	54	NA	Symptomatic	RT-PCR	Evaluated thyroid function in COVID-19 patients	80
Gong et al, 2021 (32)	Retrospective	Consecutive	China	150	81/69	69.5	NA	Symptomatic	RT-PCR	Evaluated thyroid function in COVID-19 patients	43
Güven and Gültekin, 2021 (33)	Prospective	Consecutive	Turkey	250	157/93	68	NA	Symptomatic	RT-PCR	Clinical characteristics of severely or critically ill COVID-19 patients, and patient with mild to moderate COVID-19	24
Khoo et al., 2021 (34)	Prospective	Consecutive	United Kingdom	334	271/63	66.1	16	Symptomatic	RT-PCR	Evaluated thyroid function in COVID-19 patients compared to patients without COVID-19	45
Muller et al., 2020 (36)	Prospective	Consecutive	Italy	145	89/56	67.1	14.9	Symptomatic	RT-PCR	Clinical characteristics of severely or critically ill COVID-19 patients, and patient with mild to moderate COVID-19	14
Lui et al., 2021 (35)	Prospective	Consecutive	Hong Kong	367	172/195	54	NA	Symptomatic	RT-PCR	Evaluated thyroid function in COVID-19 patients	27
Vassiliadi et al., 2021 (37)	Retrospective	Consecutive	Greece	102	76/26	59.3	18.3	Symptomatic	RT-PCR	Clinical characteristics of severely or critically ill COVID-19 patients, and patient with mild to moderate COVID-19	41
Zou et al., 2021 (40)	Retrospective	Consecutive	China	149	71/78	47	NA	Symptomatic	RT-PCR	Evaluated thyroid function in COVID-19 patients	41
Wang et al., 2021 (38)	Retrospective	Consecutive	China	84	53/31	57.3	14.5	Symptomatic	RT-PCR	Evaluated thyroid function in COVID-19 patients	52
Zhang et al., 2021 (39)	Retrospective	Consecutive	China	71	40/31	62.7	1.6	Symptomatic	RT-PCR	Evaluated thyroid function in COVID-19 patients	25

### Quality assessment and publication bias

**Figure 2:**
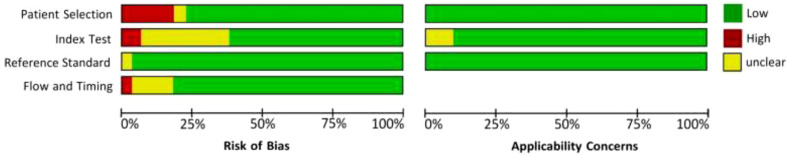
Quality assessment of studies by using QUADAS-2 tool.

In this step, we evaluated the quality assessment and ROB; in this regard, **Figure 2** shows the overall quality assessment using the QUADAS-2 tool. Based on the quality assessment results achieved from each study, we considered that the overall ROB is low to moderate. Most of the studies had a low ROB (20 studies), six had medium ROB, and four had high ROB; the primary source of bias was unclear patient selection process (i.e., missing or unclear inclusion/exclusion criteria). In addition, the flow and timing domain was judged to be high risk in five studies, and the index test domain was estimated to be high risk in nine studies.

**Figure 3:**
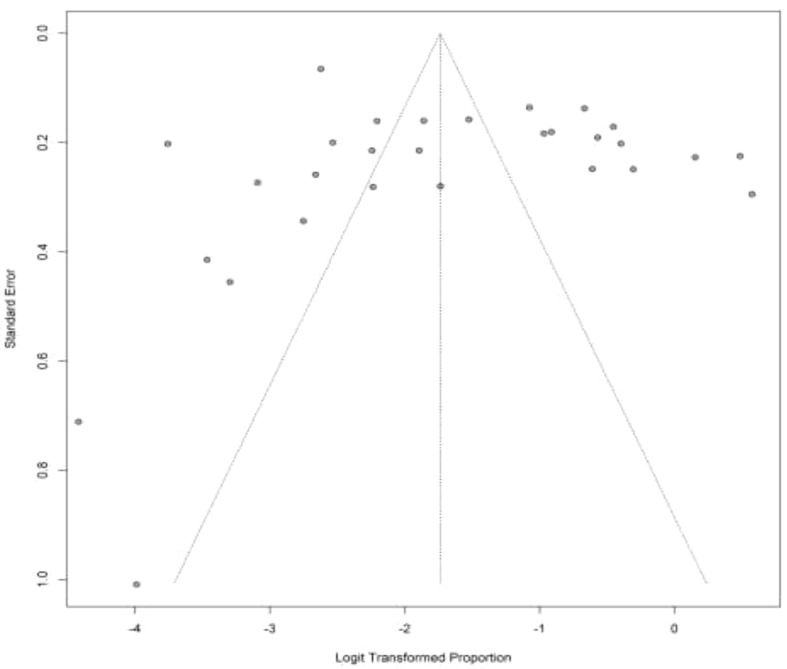
Funnel plot of enrolled studies based on abnormal TFT.

We also evaluated the potential risk of publication bias for any abnormal thyroid function test using Egger’s test method (**Figure 3**). Egger’s test revealed no risk for publication bias (P= 0.317).

### Prevalence of thyroid dysfunction in COVID-19 patients

Herein, we evaluated the prevalence of abnormal thyroid function in COVID-19 patients across all studies. Our results showed that the highest prevalence was observed in a Chinese report with a high of 64% (15), and the lowest prevalence of thyroid dysfunction was observed in a Chinese report with a prevalence of 1.2% (25) (**Table 2**). Our results showed significant high heterogeneity between Chinese studies (P<0.05) in comparison to reports originating from other countries (P>0.05). These results showed that Chinese reports could be the main cause of ROB in this section. Overall, the pooled analysis showed that the prevalence of thyroid dysfunction among 9,707 COVID-19 patients was 15% (**Table 2**).

**Table 2:** Evaluation of the prevalence of thyroid dysfunction among COVID-19 patients.

Author	Country	Total No. of patients	Prevalence, %
Lui et al., 2020 (20)	Hong Kong	191	13.1 [8.7-18.7]
Chen, T et al., 2019 (5)	China	274	17.9 [13.5-22.9]
Chen, M et al., 2020 (15)	China	50	64 [49.2-77.1]
Gao et al., 2020 (16)	China	100	15 [8.7-23.5]
Georges et al., 2020 (17)	France	433	9.9 [7.3-13.1]
van Gerwen et al., 2020 (23)	United States	3703	6.8 [6-7.6]
Cao et al., 2020 (14)	China	198	3 [1.1-6.5]
Shabrawishi et al., 2020 (21)	Saudi Arabia	150	6 [2.8-11.1]
Sisó-Almirall et al., 2020 (22)	Spain	322	4.4 [2.4-7.2]
Wang et al., 2020 (24)	China	55	1.8 [0.1-9.7]
Yan et al., 2020 (25)	China	168	1.2 [0.1-4.2]
Zhang et al., 2020 (12)	China	140	3.6 [1.2-8.1]
Almazeedi et al., 2020 (13)	Kuwait	1096	2.3 [1.5-3.4]
Lania et al., 2020 (18)	Italy	287	25.4 [20.5-30.9]
Ahn et al., 2021 (26)	South Korea	119	36.1 [27.5-45.5]
Baldelli et al., 2021 (27)	Italy	66	42.4 [30.3-55.2]
Ballesteros Vizoso et al., 2021 (28)	Spain	78	53.9 [42.2-65.2]
Beltrão et al., 2021 (29)	Brazil	245	6.5 [3.8-10.4]
Campi et al., 2021 (30)	Italy	144	38.9 [30.9-47.4]
Dutta et al., 2021 (31)	India	236	33.9 [27.9-40.3]
Gong et al, 2021 (32)	China	150	28.7 [21.6-36.6]
Güven and Gültekin, 2021 (33)	Turkey	250	9.6 [6.3-14]
Khoo et al., 2021 (34)	United Kingdom	334	13.5 [10-17.6]
Muller et al., 2020 (36)	Italy	145	9.7 [5.4-15.7]
Lui et al., 2021 (35)	Hong Kong	367	7.4 [4.9-10.5]
Vassiliadi et al., 2021 (37)	Greece	102	40.2 [30.6-50.4]
Zou et al., 2021 (40)	China	149	27.5 [20.5-35.4]
Wang et al., 2021 (38)	China	84	61.9 [50.7-72.3]
Zhang et al., 2021 (39)	China	71	35.2 [24.2-47.5]
Pooled	Worldwide	9707	15% [9.8-22.2]
I^2 = 97.1% [96.5%; 97.6%]	Cochran’s Q P< 0.001		

The exact TSH and fT3 levels were merely included in nineteen studies, among which one study was selectively conducted on low T3 syndrome (43). One study has categorized the TFT disturbances into two groups: 10 patients manifesting isolated low T3 syndrome and ten patients who presented with isolated low TSH syndrome (1). Among the remaining three studies, fT3 was lower in cases with COVID-19 compared to controls. One study noted that when categorizing covid-19 patients into mild and severe, fT3 was lower in the severe group (44). The other eleven included studies that have mentioned thyroid disorders without presenting exact TSH or fT3 values. No differentiation was indicated regarding hypo/hyperthyroidism or low T3 syndrome (36, 44–50).

### COVID-19 severity associated with thyroid function test

Among 30 studies, 23 have evaluated the role of TFT in the severity of COVID-19 directly/indirectly. Of these studies, six studies explored the mean level of TSH, fT3, and fT4 in severe and non-severe COVID-19 patients. Data obtained from fifteen studies showed that 2,841 patients experienced mild to moderate COVID-19, and 1,024 patients experienced severe critical COVID-19. Among mild to moderate COVID-19 patients, 176 patients had abnormal TFT (6.2%), and among patients who experienced severe to critical COVID-19, 213 patients had abnormal TFT (20.8%). Our results showed that the rate of abnormal TFT was significantly higher in severe to critical COVID-19 patients compared to mild to moderate COVID-19 patients.

**Figure 4:**
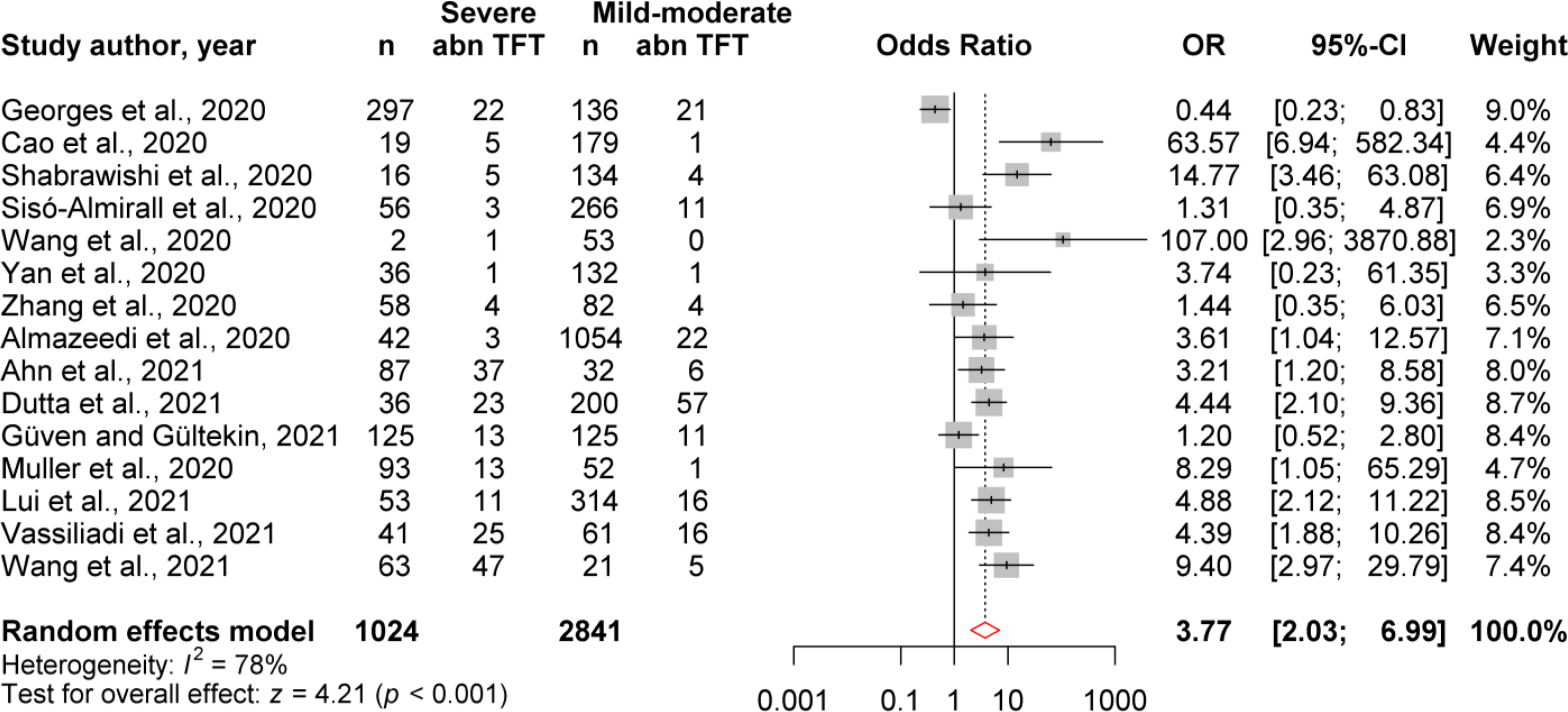
Evaluation of OR of abnormal TFT for COVID-19 severity. Experimental: patients faced severe to critical form of COVID-19, Control: patients faced mild to moderate form of COVID-19, Events: patients with abnormal TFT.

On the other hand, to complete the risk of severity, we evaluated the odds ratio (OR) of abnormal TFT in presenting severe COVID-19 (**Figure 4**). Our random effect odds ratio analysis showed that the highest OR for abnormal TFT was 107.0 (2.9, 3870), and the lowest was 0.44 (0.23, 0.83). Pooled analysis showed that the overall OR obtained from 3,865 COVID-19 patients was 3.77 (2.03, 6.99) (**Figure 4**). Thus, there is a 3.77-fold association between the abnormal TFT and the severity of COVID-19.

### The hazard ratio of TSH for COVID-19

**Figure 5:**
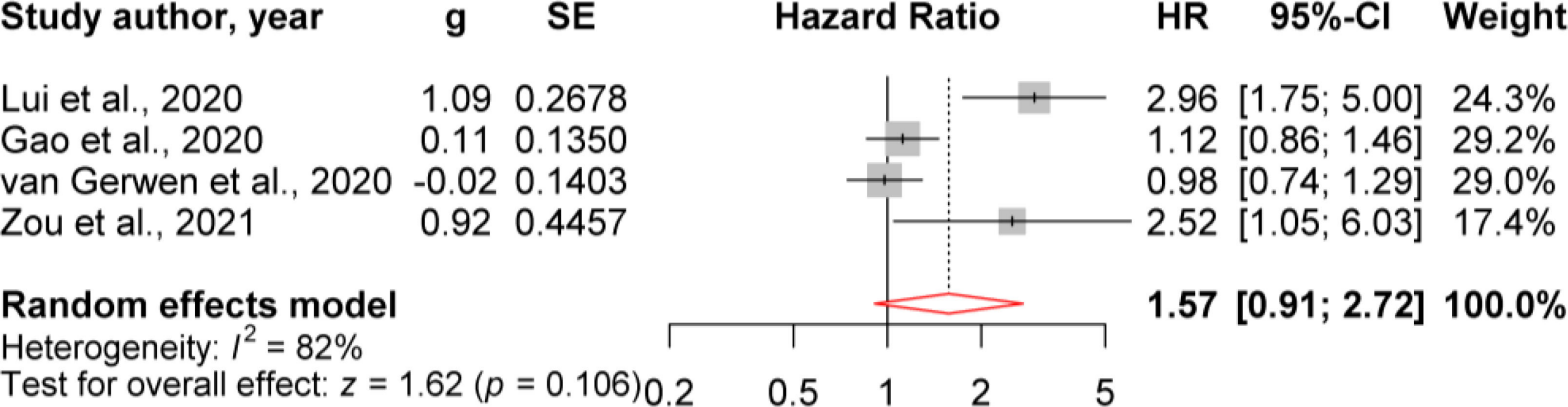
Evaluation of HR of abnormal TSH for COVID-19 severity.

In this section, we extracted the four manuscripts which have evaluated the severity in COVID-19 patients with normal/abnormal TSH levels based on hazard ratio (HR) analysis (**Figure 5**). **Figure 5** illustrates the forest plot of HR analysis of each study and the pooled HR. Our results showed that the minimum HR was 0.98 (0.75, 1.30), and the maximum HR was 2.96 (1.75, 5.00) with the random effect. The pooled HR of TSH level of COVID-19 mortality was 1.57 (0.91, 2.72). This result shows that patients with abnormal TSH had a non-significant 1.57-fold higher risk of disease severity than patients with normal TSH levels.

## Discussion

Among 30 enrolled studies, our review revealed that the ROB of the available studies is estimated low to moderate. The pooled analysis showed a high prevalence of thyroid dysfunction among patients with COVID-19 (15%). Thyroid dysfunction was associated with the severity of COVID-19, as its prevalence was 6.2% in mild to moderate cases versus 20.8% among patients with severe COVID-19. The pooled OR for the association of abnormal TFT and severe form of COVID-19 was statistically significant.

Previously published documents revealed a strong bidirectional association between thyroid disorders and COVID-19. Up to now, activation of preexisting thyroid dysfunction, hypo or hyperthyroidism and, subacute thyroiditis had been noted as sequels of COVID infection. Studies suggested that thyroid dysfunction could arise by direct insult tissue or indirectly (51, 52). One of the probable mechanism is that the thyroid gland and hypothalamic-pituitary axis could be indirectly affected by the irregular systemic inflammatory immune response triggered by SRAS-CoV-2 infection (49). Moreover, several studies are published on the thyroid gland histopathological findings in patients with COVID-19. Pathological examination of thyroid tissue showed high affinity for SRAS-CoV-2 mediated by Angiotensin-converting enzyme 2 (ACE-2) receptor (53). In addition, studies have reported interstitial lymphocytic infiltration and epithelial layer disturbance (19, 20). Importantly, histopathological studies have suggested that virus infiltration in endocrine glands can be observed in severe cases of COVID-19. Such studies have detected the viral genome and proteins inside endocrine tissues and have identified signs of apoptosis as a result of this viral invasion (54, 55), which signifies the potential for direct damage to the thyroid gland in severe COVID-19 cases.

The World Health Organization (WHO) recommendation for clinical management is not to evaluate the thyroid function in COVID-19 cases (19). However, in some trials, improvements in thyroid function have been identified in the preceding coronavirus epidemic of SARS-CoV (12, 13). Wang et al., In a particular report, showed that lower serum levels of TSH, T3, and T4 were observed in COVID-19 patients compared to normal healthy individuals (24). In addition, they observed low T3 and T4 levels in different disease phases—94% and 46% in the acute phase and 90% and 38% in the convalescent phase for T3 and T4, respectively. Similarly, Leow et al. (22) reported four (6.7%) SARS patients becoming biochemically hypothyroid three months after rehabilitation, including three with core hypothyroidism and a recent persistent lymphocytic hypothyroidism. In the three cases with core hypothyroidism, the case with primary hypothyroidism spontaneously remitted after three/nine months to lifelong T4 therapy (17). Consequently, it was discovered from the SARS outbreak that a viral infection could primarily induce low TFT levels, either on its own or in combination, through primary or secondary injuries (i.e., hypothalamic or pituitary). Moreover, the low levels of TSH and T3 may be seen as part of an adaptive condition of anti-thyroidal disease syndrome caused by a significant stress situation, especially in extreme or critically ill patients (i.e., systemic virus disease) (14, 21, 25). Additionally, evidence suggests,although, preexisting thyroid disease will not increase risk of COVID infection and complication (56), COVID could activate subclinical conditions in susceptible population or cause relapse of known condition specially Grave’s disease which is mostly temporary and does not need further management, however, as it can aggregate existing condition specially inducing thyroid storm in hyperthyroidism proper management and regular monitoring of thyroid function in this population is desirable and recommended (51, 57). As for follow up in these patients, since in most patients, thyroid dysfunction remit in three month no conclusion is proposed for further follow-up by studies and experts (51).

Low T3 syndrome, also known as the euthyroid sick syndrome (ESS) could be responsible partly for high prevalence of thyroid dysfunction in COVID-19. This syndrome is known to occur in 60 to 70% of critically ill patients regardless of the cause (58) and in different studies in admitted patients due to COVID, ESS was presented in around 30% patients(up to 64%) and were related to severity, longer hospitalization and mortality (40, 59). Similarly, studies have suggested a significant inverse correlation between the severity of illness and low serum total T3. In contrast, there was no relationship between total thyroxine or TSH levels and severity of illness and TSH levels are indicated to be within the normal range or relatively low (49). Consequently, some studies suggested evaluating free triiodothyronine (fT3) as a prognostic factor in COVID (60). Moreover, it is noteworthy that like other causes of Low T3 syndrome, thyroid function tend to go back to normal after the acute phase of the disease (20). This point is particularly remarkable since our results indicated significantly higher rates of abnormal TFTs in patients with severe to critical forms of COVID-19 compared to mild to moderate forms.

However, due to a lack of past medical history and follow-up data on these patients, no diagnostic confirmation is plausible. Moreover, some included studies that have mentioned thyroid disorders without presenting exact TSH or fT3 values. No differentiation was indicated regarding hypo/hyperthyroidism or low T3 syndrome (12–14, 17, 21–25). Therefore, no subgroup analysis was conceivable. These are particularly noteworthy since, considering the lack of follow-up data on TFTs post-discharge, we are unable to discuss our results in terms of etiological aspects. The lack of data on TFTs might be due to the fact that thyroid disorders were not initially associated with COVID-19 and were only later discussed.

On the other hand, current literature on COVID- 19 patients with thyroid dysfunction presented more evidence that SARS-CoV-2 damage targets could originate from the thyroid gland and the entire hypothalamic-pituitary-thyroid (HPT) axis and can be manifested as thyrotoxic, hypothyroidism, and nonthyroidal disease. SARS-CoV-2 can directly insult thyroid tissue and like other respiratory virus causes thyroiditis. Till now several cases of subacute thyroiditis (SAH) were documented in COVID. Among 21 reported patients with SAT, about 75% were female and was mostly presented as painful SAT with the presentation of thyrotoxicosis (61). Also, in a recently published cohort study from India, around 1.6% patients were diagnosed with SAT (62). Just like in other viral thyroiditis, in most cases SAT subsided without permanent complication (61).

Nevertheless, one point that should be noticed is that drug interactions also, might lead to TFT disturbances. For instance, Heparin-induced increase in non-essential fatty acid displaces T4 from thyroid-binding protein and causes an artefactual increase in free hormone levels. Alterations of thyroid-binding globulin can cause a corresponding change in total T4 and T3 levels (18). Corticosteroids are also known to reduce the conversion of T4 to T3, and therefore, lead to reduced T3 serum levels. This mechanism also operates regarding other common drugs used for ICU patients, including iodine and amiodarone (19). Since these drugs are commonly prescribed for severe COVID-19 cases, they can potentially be in charge of TFT alterations. Therefore, future research is required to thoroughly investigate the potential confounding role of COVID-related medications that could alter TFT levels. However, we could not address this issue in our study due to a lack of data regarding the prescribed medications in most included studies.

Our study limitations were as follows: 1- some included studies only reported the rate of patients with hypothyroidism; thus, hyperthyroidism or euthyroid diseases were missed. 2- Due to the lack of post-discharge follow-up, we were unable to analyze the progress of thyroid hormones over time. 3- We could not conclude that whether abnormal TFT could impact COVID-19 severity or COVID-19 might be severe in patients with abnormal TFT. 4- We were unable to run a meta-regression between the serum levels of TSH, T3, and T4 due to a lack of data, and a mere OR analysis was conducted. Nonetheless, our review has generated the largest available data regarding the association of COVID-19 and thyroid dysfunction.

Last but not least, several studies have suggested a potential causal association between COVID-19 infection and TFT abnormalities; however, the results of our meta-analysis do not focus on causality and do not establish a causal association between COVID-19 and thyroid dysfunction, mainly due to significant heterogeneity in populations, low sample sizes, and lack of investigation of a direct causal role for COVID-19 in most included studies. But what is apparent is that thyroid dysfunction is accompanied with more severe form of COVID thus monitoring TFT seems to be beneficial specially in some populations including patients with preexisting thyroid condition, in severe form of infection, and in patients with manifestation of viral thyroiditis (neck pain). Likewise, regarding to the new published article inventing a scoring system for thyroid dysfunction in COVID patient with five parameters including symptoms, presence of ischemic heart disease/congestive heart failure and abnormal laboratory finding (lymphocyte count, C-reactive protein, and SARS-CoV-2 cycle threshold values) should be considered for TFT evaluation (63).

## Conclusion

Our study showed that the prevalence of thyroid dysfunction among patients with COVID-19 was as high as 15%. In addition, there is a 3.77-fold association between abnormal TFT and the severity of COVID-19. Lastly, patients with TSH lower than ~1 had a 1.57-fold higher risk of disease severity and poor outcomes than patients with normal TSH levels. Further studies are required to understand the prognostic significance of thyroid dysfunction in severe COVID-19 and investigate therapeutic approaches to reduce poor outcomes associated with this clinical condition.

## Author contributions

MD, MRN, HS, and MN participated in the design of the review, acquired the documents and made a discussion, edited the manuscript, supervised the study and wrote the paper, collected the data and contributed to figures and tables. All authors contributed to the article and approved the submitted version.

## Conflict of interest

The authors declare that the research was conducted in the absence of any commercial or financial relationships that could be construed as a potential conflict of interest.

## Publisher’s note

All claims expressed in this article are solely those of the authors and do not necessarily represent those of their affiliated organizations, or those of the publisher, the editors and the reviewers. Any product that may be evaluated in this article, or claim that may be made by its manufacturer, is not guaranteed or endorsed by the publisher.
